# *Genes and Environment*: reflections on its journey, past and future

**DOI:** 10.1186/s41021-025-00333-z

**Published:** 2025-05-21

**Authors:** Takashi Yagi

**Affiliations:** Graduate School of Science, Osaka Metropolitan University, 1-2 Gakuen-cho, Naka-ku, Sakai, Osaka, Japan

**Keywords:** JEMS official journal, Journal development, Internationalization of JEMS and journal, Research dissemination

## Abstract

This article outlines the history and development of *Genes and Environment*, the official journal of the Japanese Environmental Mutagen and Genome Society (JEMS). In the 1970s, there was growing concern about the mutagenicity of chemical substances, leading to the establishment of JEMS. The society began publishing its journal, *Environmental Mutagen Research*, and renamed *Genes and Environment* in 2006 to focus on gene-environment interactions and promote international collaboration. The journal transitioned to free-access and started publishing in English to expand its reach globally.

From 2012, the journal partnered with BioMed Central (BMC) to become an open-access publication, leading to its inclusion in Scopus, PubMed, and SCIE, and an improvement in its CiteScore and Impact Factor. JEMS also sought funding from Japan’s Grants-in-Aid for Scientific Research (KAKENHI) to support international dissemination of research.

Despite progress, challenges remain, such as limited submissions from certain regions and a need for greater global recognition. To further internationalize JEMS, efforts are being made to elevate the quality of research and broaden membership diversity, with a focus on making JEMS’ activities and publications more accessible to the global scientific community.

## Introduction — the founding of the Japanese Environmental Mutagen Society (JEMS)

Mutagenicity or genotoxicity is a relatively new field of toxicology that emerged after 1970 [1]. It was known before the 1950s that certain chemicals, such as alkylating agents, could induce mutations. However, at the time, these phenomena were considered specific effects of particular chemicals and were largely confined to the research domain of geneticists. After World War II, as studies on the genetic effects of radiation — particularly the aftereffects of atomic bomb exposure — progressed, concerns gradually grew about the potential genetic harm of chemical substances to humans.

In the 1960s, the idea that this issue should be evaluated for all chemical substances gained momentum, leading to the initiation of fundamental research in public research institutions in Europe and the United States. The U.S. government recognized the importance of mutagenicity testing for pesticides and pharmaceuticals, which was proposed in 1969 and 1970, respectively. In 1969, the Environmental Mutagen Society (EMS) was established in the United States, followed by the founding of the European EMS in 1970. These global research trends of that period are introduced by Kikuchi [1] and Sugimura [2].

In Japan, after World War II, fundamental research on the genetic effects of radiation was conducted at universities and major institutions, using various organisms and mammalian cultured cells, as Japan was a country affected by atomic bomb exposure. However, research on the effects of chemical substances was minimal.

A turning point in this situation came in 1968. At the 12th International Congress of Genetics held in Tokyo that year, the mutagenicity of environmental chemicals emerged as a new research topic. Dr. Yataro Tajima of the National Institute of Genetics strongly recognized the need to establish an organized research framework for studying the mutagenicity of chemical substances in Japan. Having conducted extensive research in radiation genetics for many years, he felt a deep sense of urgency regarding the mutagenic potential of chemical substances as well.

In 1970, Dr. Tajima organized a special research team under the Ministry of Education, launching a systematic study titled “Research on the Mutagenicity of Chemical Substances.” This research group became the foundation for the establishment of the Japanese Environmental Mutagen Society (JEMS). The founding general meeting of JEMS was held in October 1972 in Tokyo, under the title “1st Conference on Environmental Mutagen Research.”

In September 1973, the 2nd JEMS research meeting was held at the National Institute of Genetics in Mishima. Many presentations at the meeting were related to AF-2 (furylfuramide), a food additive that was commonly used as a food preservative at the time. The mutagenicity revealed by various tests brought AF-2 to the forefront as a significant topic of discussion around the time JEMS was founded [1]. Growing concerns about the safety of widely used food additives became a major social issue.

## Publication of the journal *Environmental Mutagen Research*

**Fig. 1:**
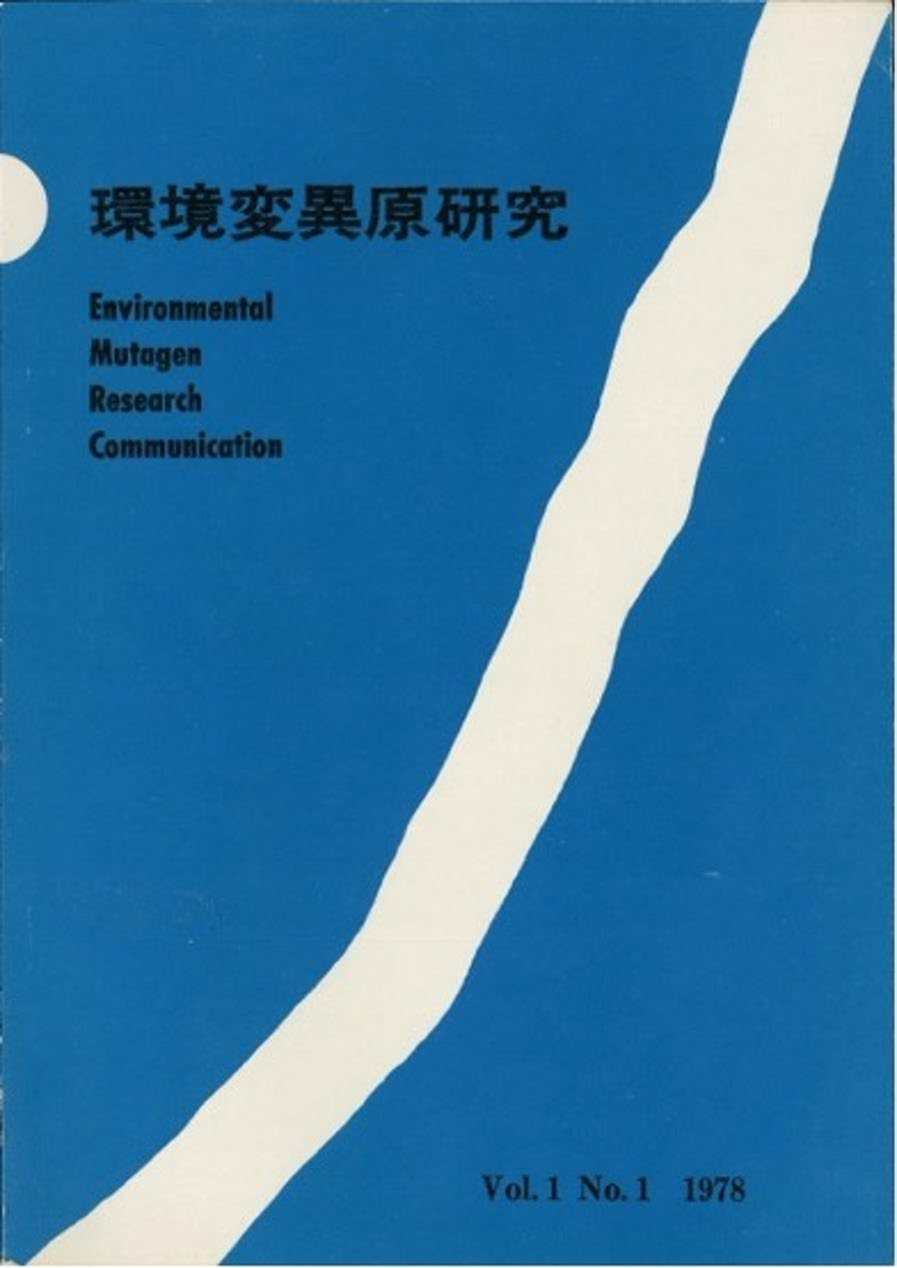
Cover of *Environmental Mutagen Research Communication*

JEMS first published its academic journal, *Environmental Mutagen Research Communication*, in 1978 (Vol. 1) (Fig. 1). The journal’s name was changed to *Environmental Mutagen Research Communications* in 1981 (Vol. 3), and later to *Environmental Mutagen Research* in 1998 (Vol. 20). The journal aimed to present original research papers and review articles, introduce researchers’ studies, and facilitate information exchange among researchers. Most of the papers in these issues were written in Japanese. The journals are now available for viewing at the National Cancer Research Center Library from Vol. 1 (1978) to Vol. 23 (2001) [3]. Papers from Vol. 23 (2001) to Vol. 25 (2003) and from Vol. 25 (2003) to Vol. 27 (2005) can be downloaded from the JEMS and J-STAGE websites, respectively [4].

**Fig. 2:**
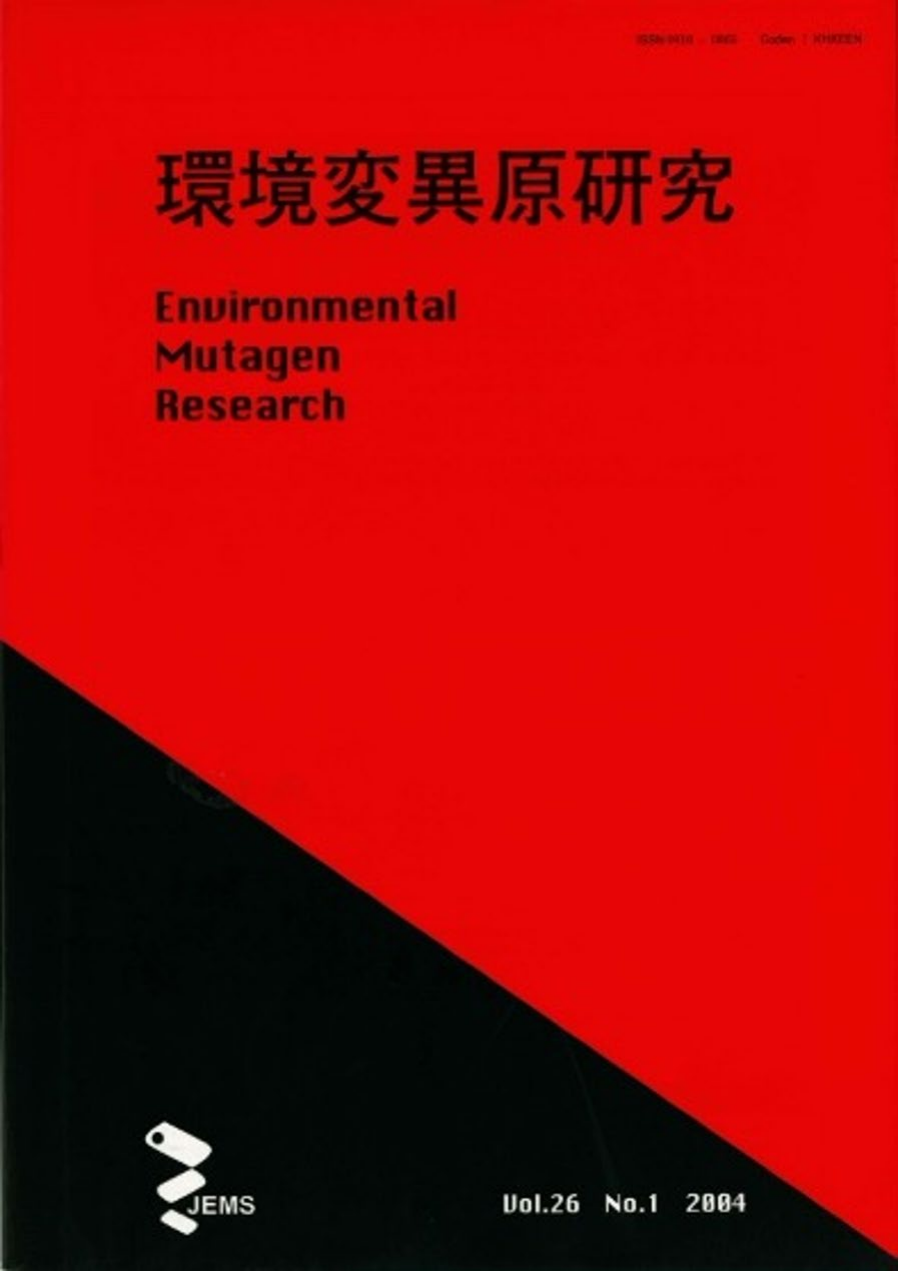
Cover of *Environmental Mutagen Research*

In 1995, the design of the journal cover was publicly solicited by Dr. Motoi Ishidate, the Editor-in-Chief at the time, and the red-and-black design by Dr. Chie Furihata was selected. In 2003, the JEMS logo was also publicly solicited by the joint Editorial and Public Relations Committee, and the design by Dr. Yutaka Ishii was chosen and featured on the bottom left of the journal cover (Fig. 2).

## Renaming the journal to *Genes and Environment* and transitioning to free access

Over time, understanding the interactions among chemicals, genes, and the environment has become crucial for developing cancer prevention measures and advancing the regulation of chemical substances. The regulation of genotoxic pharmaceuticals, pesticides, and food additives remains a major concern in genetic toxicology. A mechanistic understanding of how cells respond to environmental threats and protect their genetic integrity significantly contributes to establishing fundamental policies for risk assessment. Recognizing the importance of gene-environment interactions in disease etiology, JEMS renamed its journal *Genes and Environment* [5].

To enhance communication and collaboration with scientists outside Japan, particularly in other Asian countries, JEMS decided to publish the journal entirely in English starting in 2006. *Genes and Environment* covers a broad range of fields, including environmental mutagenesis, environmental genomics, molecular epidemiology, and genetic toxicology, and welcomes manuscript submissions from both JEMS members and non-members [5]. Dr. Minako Nagao served as the first Editor-in-Chief of *Genes and Environment* and played a key role in establishing international standards for the journal, including submission guidelines, the editorial board, and the peer review system.

**Fig. 3:**
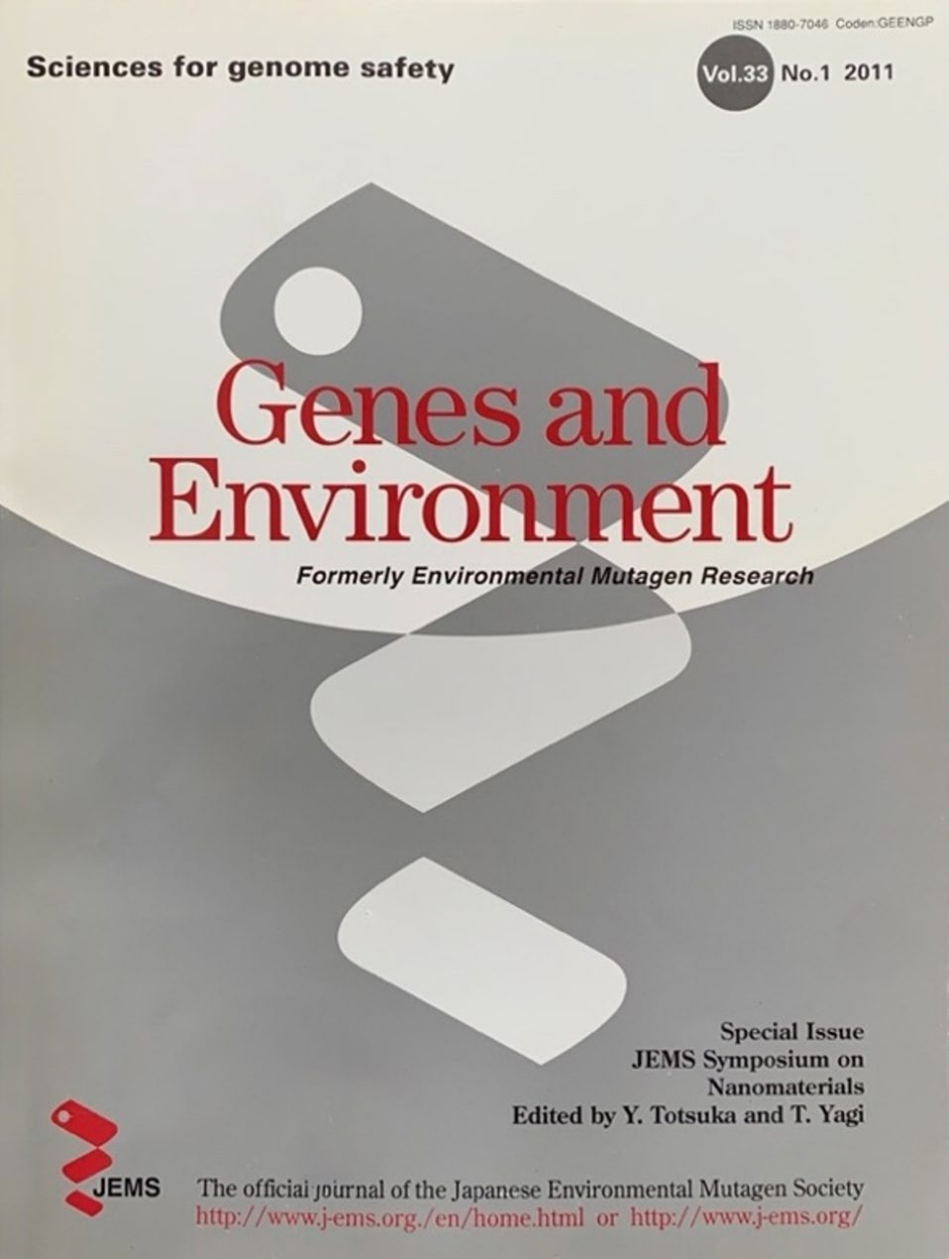
Cover of *Genes and Environment* (print edition)

The journal was published four times a year in both print and electronic formats. The cover design of the print edition was selected by the JEMS board of councilors from designs proposed by the editorial committee (Fig. 3). The contents are freely available for download from J-STAGE [6] and can also be accessed through the JEMS website [7]. Thus, *Genes and Environment* became a free-access journal, allowing anyone worldwide to read its papers free of charge.

## Publication of *Genes and Environment* by BioMed Central and transitioning to open access

After Dr. Nagao’s outstanding editorial leadership for six years, the role of Editor-in-Chief was passed to me in 2012. At that time, JEMS faced two major challenges regarding journal publication. The first issue was the high cost of publishing the printed version of the journal, which amounted to nearly 3 million yen annually, placing a heavy financial burden on JEMS. The second issue was the significant workload of the Editor-in-Chief in managing the publication process for submitted manuscripts, which interfered with their primary duties as a university professor.

While exploring solutions to these issues, I received a proposal from BioMed Central (BMC) Japan to transition *Genes and Environment* to an open-access journal using their platform. At the time, I believed that JEMS members would never accept the idea of authors paying an article processing charge (APC) to publish their papers. However, BMC insisted that this was the global trend, and indeed, open-access journals have since become the mainstream. I invited representatives from BMC, Elsevier, and Wiley to present on the publication of open-access journals at the JEMS Editorial Committee.

In May 2014, after discussions among Editorial Committee members and Council members, JEMS selected BMC as the journal’s open-access publisher [8, 9]. Since 2015 (Vol. 37), articles in *Genes and Environment* have been accessible via the journal’s website on BMC [10], while previous issues remain available on the J-STAGE website [6].

Ms. Natsu Ishii, the sales representative from BMC Japan, provided detailed explanations on multiple occasions regarding the transition to the BMC platform, the contract between JEMS and BMC, and the adoption of Editorial Manager (EM) as the journal’s online submission and manuscript management system. Additionally, Ms. Noriko Lebowits and later Ms. Masayo Kobayashi, who have overseen *Genes and Environment*, attended every JEMS Editorial Board meeting, continuously offering advice on the journal’s current status and future development.

BMC Japan also facilitated a three-party meeting with BMC’s UK headquarters and its editorial office in Germany. Furthermore, representatives from the London headquarters visited Japan twice: first, to hold a seminar on open-access journals in Tokyo, and later, to deliver remarks at the JEMS Council meeting during the JEMS Annual Conference in Fukuoka in November 2015. BMC Japan set up a promotional booth for *Genes and Environment* at the Fukuoka Conference and also supported JEMS in establishing an exhibition booth at the International EMS Conference in Korea in November 2017. I am grateful for the extensive support that the BMC Japan staff has provided to JEMS over the years.

It is worth noting that BMC was a part of Springer, which merged with Nature Publishing Group in 2015.

## Indexed in Scopus, PubMed, and SCIE: Journal Impact Factor announced

*Genes and Environment* has been indexed in Scopus since its initial publication following JEMS’s application for indexing [11]. In July 2016, the journal was further indexed in PubMed based on BMC’s recommendation, significantly expanding its readership [12]. These databases have enhanced the journal’s international recognition and increased citations in other international journals. However, submissions from overseas remained limited, prompting the JEMS Editorial Committee to seek new strategies to attract global contributions.

Many scientists worldwide consider the Impact Factor (IF) provided by Clarivate Analytics’ Science Citation Index Expanded (SCIE) a key indicator of a journal’s quality [13]. Recognizing this, the JEMS Editorial Board took steps to have *Genes and Environment* included in SCIE. In June 2018, Dr. Takehiko Nohmi and Dr. Masami Yamada visited the Japan office of Clarivate Analytics, where Mr. Mikihiko Nimura provided guidance on the requirements for SCIE inclusion. In the application, we stressed the importance of communication among Asian scientists because of the increased environmental problems, such as air pollution and food insecurity, in the area.

Following Dr. Yamada’s application for SCIE indexing in July 2018, Clarivate Analytics provided an exceptionally swift response, notifying JEMS in November 2019 that the journal had been accepted for inclusion. Additionally, BMC officially announced that *Genes and Environment* would receive the IF starting in 2020. The journal’s first IF, based on 2019 data, was 1.872.

## Application and award of the Grant-in-Aid for Scientific Research

With *Genes and Environment* becoming an open-access journal, JEMS’s financial situation improved through authors’ APC payments. However, the funding was still insufficient. The next goal of the JEMS Editorial Committee was to secure a publication grant from the Grant-in-Aid for Scientific Research (KAKENHI) of the Japan Society for the Promotion of Science (JSPS) [14].

In 2015, a new KAKENHI grant category, “Open Access Support,” was introduced. Although *Genes and Environment* had already transitioned to open access the previous year, we applied under this program with the proposal title: “Enhancing the International Dissemination of Research and Improving the Quality of Society Journals through Full Open Access.” I drafted the application, which was then reviewed by Dr. Nohmi and Dr. Yasunobu Aoki, the JEMS president at the time. Thanks to their thorough revisions, JSPS invited us for an interview. I did my best during the five-minute presentation and subsequent Q&A session, but our application was ultimately rejected. That year, only two societies were selected: the Physical Society of Japan and a large medical society. I realized that it is not easy for a small society like JEMS to secure this grant.

In October 2015, we applied again for the 2016 KAKENHI grant under a different category, “Enhancement of International Dissemination of Information,” with the same proposal title, but it was again unsuccessful.

Determined to succeed, the JEMS Editorial Committee formed a project team in the summer of 2016 to apply for the 2017 KAKENHI grant. The team included Dr. Nohmi, Dr. Aoki, Dr. Yamada, Dr. Masanobu Kawanishi, Dr. Yoshifumi Uno, and myself. In September, Dr. Uno, Dr. Nohmi, Dr. Yamada, and I visited JSPS to seek advice on securing funding. Additionally, we reviewed application documents kindly provided by two academic societies that had previously received KAKENHI funding. With this insight, the team refined my draft into a polished and well-structured proposal.

We applied under the category “Enhancement of International Dissemination of Research Results” with the proposal title: “Research on Environmental Mutagens and Carcinogens in Asia, Disseminated from Japan”. On April 1, 2017, JEMS received notification from JSPS that our application had been accepted—it almost felt like an April Fool’s joke.

The JEMS 2017 KAKENHI project included waiving APC for *Genes and Environment* papers submitted from Asian countries, increasing the number of overseas editorial board members, promoting the journal at International EMS Conferences (ICEM), and inviting Asian scientists to the JEMS Annual Conference to encourage submissions from the region. As part of these efforts, JEMS conducted promotional activities at ICEM in Korea (November 2017) and at the All India Congress of Cytology and Genetics (AICCG) in India (January 2018). Additionally, a *Genes and Environment* Editorial Board Meeting was held with invited foreign board members in Korea, as well as at the JEMS Annual Conference in Kyoto (2018).

In 2018, I handed over the role of Editor-in-Chief to Dr. Yamada [15]. In 2021, JEMS was renamed the Japanese Environmental Mutagen and Genome Society following the renaming of its US and European counterpart societies [16]. JEMS continued its *Genes and Environment* promotion efforts at ICEM in Canada (2022), and in the same year, the JEMS 2017 KAKENHI project successfully concluded.

## Current status and future challenges of *Genes and Environment*

One year before the conclusion of the 2017 KAKENHI project, the JEMS 2022 KAKENHI application project team, led by Dr. Yamada, began preparing the next application. The major team members were Dr. Yamada, Dr. Kawanishi, Dr. Takamura, and Dr. Nohmi. They applied again under the category of “Enhancement of International Dissemination of Research Results,” with the proposal titled “Advancement and Deepening of Environmental Mutagen Genome Research and Carcinogenesis Studies in Asia: Information Dissemination from Japan and Strengthening of Its Framework.” A key feature of the project is the creation of short films demonstrating experimental protocols for young Asian researchers. The application was accepted on April 1, 2022, and the project is currently in progress.

Since the journal’s name changed from *Environmental Mutagen Research* to *Genes and Environment*, it has shown steady growth as an international publication. This growth is evident through its inclusion in Scopus, PubMed, and SCIE, the acquisition of a CiteScore and Impact Factor (IF), and two successful KAKENHI grants. The journal’s CiteScore increased from 3.6 in 2018 to 4.0 in 2023, and its IF rose from 1.87 in 2019 to 2.7 in 2023. In 2006, there were almost no unsolicited submissions to *Genes and Environment* from overseas. By 2023, however, 32% of the published articles originated from overseas contributors. The acceptance rate for submitted papers has also declined over time, from 97% in 2008 to 75% in 2016 and 33% in 2023, suggesting the increased severity of accepting manuscripts for publication in this journal.

Since 2015, the Editorial Committee has annually selected and awarded the Best Paper from the articles published in the previous year. This initiative encourages researchers to submit high-quality papers to *Genes and Environment*, which is expected to lead to an increase in citations from other papers.

However, some issues with *Genes and Environment* still remain. Most submissions from overseas come from China, Iran, and India, while submissions from South Korea, Taiwan, and Southeast Asian countries are few. Submissions from Europe, Oceania, and the Americas are extremely rare. This indicates that *Genes and Environment* has not yet grown into a key journal in Asia, nor has it achieved full international recognition. Since JEMS played a central role in establishing the Asian Association of Environmental Mutagen Societies (AAEMS), it is crucial first to increase the journal’s recognition among AAEMS member countries. Furthermore, efforts such as setting up a *Genes and Environment* booth at ACEM and ICEM, distributing leaflets, organizing mini-seminars, and holding Editorial Board meetings will be necessary.

JEMS has received KAKENHI funding for ten years, but it is important to remember that the purpose of this support is not to subsidize the publication costs of *Genes and Environment*, but rather to enhance the international dissemination of research results. Efforts must be made to share Asia’s environmental mutagen research with the world through means other than solely publishing *Genes and Environment*. Additionally, there is no guarantee that JEMS will continue to receive KAKENHI funding in the future. It is essential to consider strategies now to sustain the publication of *Genes and Environment* without KAKENHI support.

Finally, let us consider what is necessary to truly internationalize *Genes and Environment*. First, JEMS members must enhance their own research standards and publish their findings in *Genes and Environment*. Second, the internationalization of JEMS itself is essential. JEMS membership should not be limited to Japanese researchers; rather, it should be open to researchers worldwide, similar to the US and European EMGS. The JEMS Annual Meeting should be conducted entirely in English to allow participation from researchers of any nationality. KAKENHI funding could be used to partially support travel expenses for participants from abroad. Furthermore, JEMS announcements and *Jems News* should be published in English to reach international members.

As the second Editor-in-Chief of *Genes and Environment* and a recipient of the Honorary Membership awarded last year, I offer these reflections as my message to the current members of JEMS.

## Data Availability

No datasets were generated or analysed during the current study.
